# Metformin Rescues the MG63 Osteoblasts against the Effect of High Glucose on Proliferation

**DOI:** 10.1155/2014/453940

**Published:** 2014-04-09

**Authors:** Xinyu Shao, Xiaojun Cao, Ge Song, Yuan Zhao, Bimin Shi

**Affiliations:** ^1^Department of Endocrinology and Metabolism, The First Affiliated Hospital of Soochow University, 188 Shizi Street, Suzhou, Jiangsu 215006, China; ^2^Department of Endocrinology and Metabolism, Zhangjiagang First People's Hospital, Zhangjiagang, Jiangsu 215600, China

## Abstract

*Aims*. To study the proliferation of osteoblasts and genes expression under normal glucose, high glucose, and metformin (Met). *Methods*. MG63 osteoblast-like cells were cultured in osteogenic medium supplemented with normal glucose (glucose 5.5 mmol/L) or high glucose (glucose 16.7 mmol/L) and metformin + high glucose (Met 300 **μ**mol/L + glucose 16.7 mmol/L). Proliferation was detected with CCK-8 assay at days 1, 3, and 7. Real-time PCR and Western blot were performed to compare the expression of collagen I (Col I), osteocalcin (OCN), osteoprotegerin (OPG), receptor activator for NF-**κ**B ligand (RANKL), and metal matrix proteinases 1 and 2 (MMP1, MMP2). Alkaline phosphatase (ALP) activity was also detected at days 6, 12, and 18. *Results*. Exposure to high glucose inhibited the proliferation of osteoblasts (*P* < 0.05), with suppressed OCN and OPG. Meanwhile, Col I, RANKL, MMP1, and MMP2 were unaffected. Metformin attenuated the suppression on proliferation with increased expression of Col I, OCN, and OPG, meanwhile suppressing MMP1 and MMP2. High glucose lowered the intracellular ALP, while metformin raised it. Metformin attenuated the downregulation of ALP completely at day 6, partly at day 12, but not at day 18. *Conclusions*. Metformin attenuated the suppression effect of high glucose to the osteoblast proliferation and gene expression, more prominently in earlier stage.

## 1. Introduction

Hyperglycemia is one of the main causes of osteoporosis, considering the huge population of diabetes mellitus (DM) in the world [1]. According to surveys around the world, both type 1 and type 2 diabetic patients had higher prevalence of osteopenia or osteoporosis [2, 3]. Logically, correction of hyperglycemia became one of the most reasonable approaches people can find to improve the bone mass. Among the diversified oral hypoglycemic agents (OHA) for type 2 diabetic patients, metformin was defined as the first-choice according to the ADA/EASD guidelines [4]. Recently, metformin was also found to be protective to the bone loss, with an increased bone density in diabetic animals [5]. But the subtle mechanisms of this protection, whether independent of the blood glucose control, and the detailed changes in the osteoblast differentiation and maturation are still to be elucidated. Some osteogenic genes were proved to be related to osteoblast differentiation, for example, collagen 1 alpha (Col1*α*), osteocalcin (OCN), alkaline phosphatase (ALP), osteoprotegerin (OPG), and receptor activator for NF-*κ*B ligand (RANKL) [6–9]. Besides, matrix metalloproteinases (MMPs), a series of zinc-dependent endopeptidases being capable of degrading most extracellular matrix proteins, are also reported to be related to the differentiation of osteoblast [10]. How metformin affects osteoblasts is undetermined. In the present study we explored how high glucose and metformin treatment influence the proliferation and related gene expression.

## 2. Materials and Methods

Metformin, ascorbic acid, and dexamethasone were purchased from Sigma (USA). CCK-8 proliferation assay kit was from Dojindo Bio Co., Kumamoto, Japan. TRIzol nucleotide extraction kit was from Invitrogen, NY, USA. Protein quantification kit with BCA standard was from Nanjing Jiancheng Biology Corp., Nanjing, China. RT-PCR kit was from TaKaRa, Tokyo, Japan. MG63 osteoblastic cell line was kindly donated by Professor Qin SHI, Bone Research Lab, the First Affiliated Hospital of Soochow University.

### 2.1. Cell Culture

Human osteoblast-like cells MG63 were cultured in DMEM medium, supplemented with 10% fetal calf serum, 100 U/mL penicillin, and 100**μ**g/mL streptomycin at 37°C in 95% humidified air with 5% CO_2_. The medium was replaced every 3 days, and confluent cells were digested with 0.25% trypsin and split at a rate of 1 : 2–1 : 3.

MG63 cells were grouped into four: control (Ctrl, original DMEM medium), normal glucose group (NG, 5.5 mmol/L glucose), high glucose group (HG, 16.7 mmol/L glucose), and metformin treatment group (HG + Met, 16.7 mmol/L glucose plus 300 *μ*mol/L metformin).

### 2.2. Proliferation Analysis

Cell proliferation was analyzed using Cell Counting Kit-8 (CCK-8) assays. MG63 human osteoblast-like cells were plated in 96-well plates (1 × 10^3^ cells/well). Cells were then incubated at 37°C for the next 7 days with ctrl, NG, HG, and HG + Met mediums. At day 1, day 3, and day 7 after seeding of the cells, CCK8 assay was performed according to the manufacturer's instructions. The optical density (OD) for each well was measured at 450 nm using a 96-well plate reader. Three replicate wells were used for each analysis and at least three independent experiments were performed. The cell proliferation rate was calculated according to the following equation provided by the manufacturer: the cell proliferation rate = [(OD experiment − OD blank)/(OD control − OD blank)] × 100% [11].

### 2.3. Quantitative RT-PCR of Osteoblast Related Genes

Primers were manufactured as Table 1 by Shanghai Sango Inc. (China). MG63 cells of four different treatments were induced with osteogenic medium (50 mg/L ascorbic acid, 10 mmol/L *β*-glycerol phosphate disodium, and 100 nmol/L dexamethasone). Cells were harvested at day 3 and day 7, and total RNA was extracted with TRIzol method according to the protocols recommended by the manufacturer. The total RNA concentration and quantity were assessed by absorbance at 260 nm. *β*-actin was designed as inner control of RT-PCR. Products were analyzed using real-time quantitative PCR and TaqMan probes. Real-time PCR was done in triplicate using the Applied Biosystems 7500 (NJ, USA).

### 2.4. Protein Extraction and Western Blot

MG63 osteoblasts were harvested and rinsed with cold PBS. Cells were lysed and homogenized. Then the lyastes were spined at 14000 rpm for 10 min at 4°C. Protein concentration of total protein extracted was determined by the BCA Protein Assay (Pierce Chemicals Co., Rockford, IL, USA). The protein was separated by SDS-polyacrylamide gel electrophoresis and transferred to PVDF membrane (Millipore, MA, USA) using a semidry transfer apparatus. Membrane was probed with anti-MMP1, anti-MMP2, anti-RANKL, anti-OPG (Bioworld, GA, USA), anticollagen I, anti-OCN antibody (Abcam, MA, USA), and anti-GAPDH inner control antibody (Sigma, MO, USA). And then the blots were incubated with horseradish peroxidase-conjugated secondary antibodies at room temperature for 1 h. The immune complexes were visualized with a chemiluminescence detection system (Amersham Bioscience, NJ, USA).

### 2.5. Determination of Alkaline Phosphatase (ALP)

Cells were plated in 6-well plates (1 × 10^5^ cells/well). Four groups as described above were induced with osteogenic medium mentioned above. Cells were harvested and lysed at days 6, 12, and 18. Supernatant was collected after centrifuge at 2000 r/min for 5 min. Intracellular ALP activity was measured with automatic biochemical analyzer, with BCA adjusting for the intracellular protein content.

### 2.6. Statistics

All the numeric parameters were recorded as mean ± standard deviation. The Statistical Package for Social Sciences (SPSS) 10.0 software was used for ANOVA. A *P* value <0.05 was considered statistically significant.

## 3. Results

**Figure 1:**
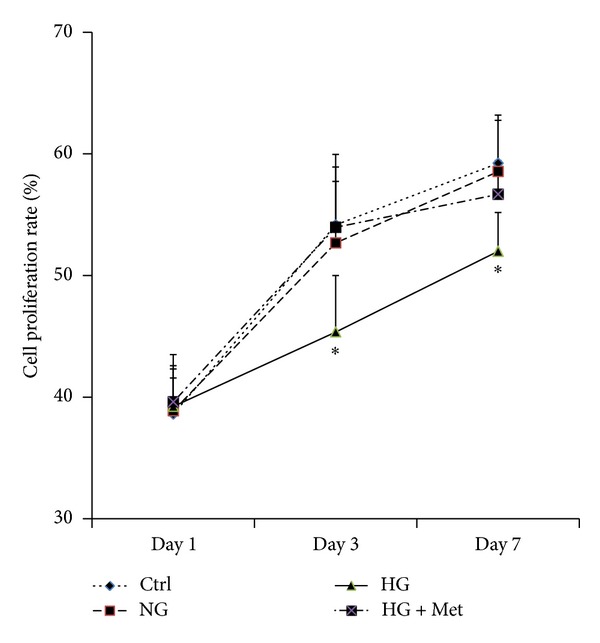
Treatment with high glucose significantly inhibited the proliferation of MG63 cells. And metformin could rescue this effect. Cell proliferation was tested by CCK8 assay as described in the methods at day 1, day 3, and day 7. Data represent mean ± SD of three separate experiments. Comparison among normal glucose (NG), high glucose (HG), and metformin treatment plus high glucose (HG + Met), *P < 0.05 HG versus ctrl, NG, HG + Met.

**Figure 2:**
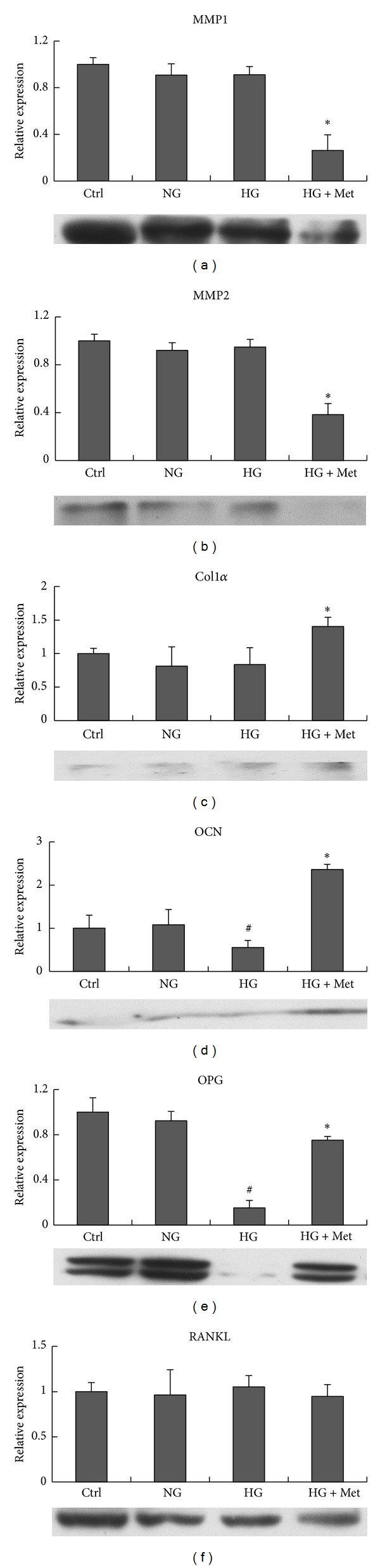
Results of real-time PCR and Western blot. MMP1 (a), MMP2 (b), Col1*α* (c), OCN (d), OPG (e), RANKL (f) mRNA expression and protein of MG63 cells under control DMEM (ctrl), normal glucose (NG), high glucose (HG), and metformin treatment + high glucose (HG + Met). *P < 0.05, HG + Met versus ctrl, NG (5.5 mmol/L), and HG (16.7 mmol/L); **P < 0.05, HG + Met versus with HG (16.7 mmol/L); ^#^ *P* < 0.05, HG (16.7 mmol/L) versus ctrl and NG (5.5 mmol/L).

**Figure 3:**
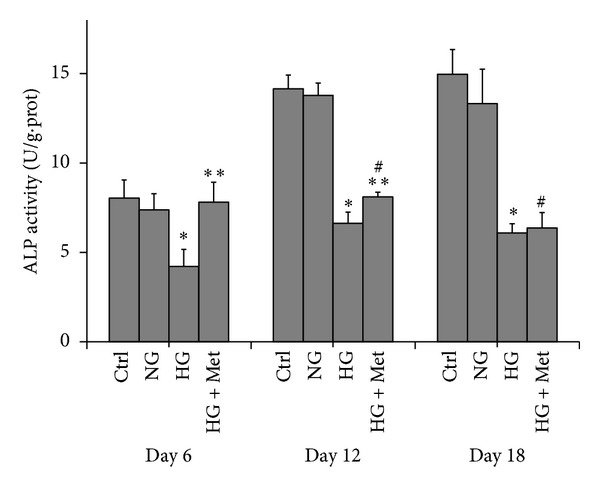
ALP activity (unit/gram of protein) of MG63 cells at days 6, 12, and 18 exposed to normal glucose (NG), high glucose (HG), and metformin (300 *μ*mol/L) treatment plus high glucose (HG + Met). Data was compared among cells at each timepoint. *P < 0.05, HG (16.7 mmol/L) versus ctrl and NG (5.5 mmol/L). **P < 0.05, HG + Met versus HG (16.7 mmol/L). ^#^ *P* < 0.05, HG + Met versus Ctrl and NG (5.5 mmol/L).

1. To assess the influence of normal or high glucose level and metformin exposure on cell proliferation of osteoblasts, MG63 cells were cultured in conditions described above, and CCK-8 assay was performed at day 1, day 3, and day 7. As shown in Figure 1, CCK-8 assay was done on MG63 osteoblast-like cells cultured with osteogenic induction mediums supplemented with different concentrations of glucose. At day 1, all the CCK-8 values and proliferation were similar. After 3 and 7 days of exposure of glucose with or without metformin, MG63 cells cultured with high glucose (Glu 16.7 mmol/L) had the lower proliferation than those with normal glucose. Metformin seemed to rescue the suppression of proliferation resulting from high glucose, both at day 3 and day 7.
2. To quantify the expression of osteogenesis-related genes, real-time PCR and Western blot were performed with the MG63 cells osteoblast-like cells cultured in osteogenic mediums supplemented with different concentrations of glucose, and metformin (300 *μ*mol/L) was also added to high glucose medium (16.7 mmol/L).  Figure 2 showed the real-time PCR and Western blot results of MG63 osteoblast-like cells cultured in different conditions mentioned previously. The data revealed that the expressions of OCN (Figure 2(d)) and OPG (Figure 2(e)) were suppressed when cultured with high glucose level of 16.7 mmol/L than those with normal glucose level of 5.5 mmol/L and control medium (*P* < 0.05), while those of MMP1 (Figure 2(a)), MMP2 (Figure 2(b)), Col1*α* (Figure 2(c)), or RANKL (Figure 2(f)) were not affected by high glucose. In addition, MMP1 and MMP2 mRNA and protein production were significantly suppressed by 300 *μ*mol/L metformin (HG + Met), not by the change of glucose level alone. It is interesting that metformin promoted the expression of OCN to the extent that is even higher than the cells cultured in normal glucose medium (Figure 2(d)). Metformin also suppressed the expression of MMP1 (decreased by 0.74-fold) and MMP2 (decreased by 0.62-fold).
3. To evaluate the function of the osteoblast, MG63 osteoblast-like cells were inducted with osteogenic mediums for 6, 12, and 18 days. Alkaline phosphatase (ALP) in these cells was detected with an automatic biochemical analyzer. As shown in Figure 3, when exposed to high glucose for 6 days, 12 days, and 18 days, MG63 cells have lower ALP activity compared with those exposed to normal glucose. In comparison, metformin could attenuate the downregulation completely at day 6, partly at day 12, but not at day 18.

## 4. Discussion

It is well-accepted that diabetes mellitus (DM) could increase the risk of osteoporosis and fragility fractures [2, 3]. As the most commonly used oral antidiabetic drug, metformin was reported to be protective against bone loss [5]. As we know, osteoblast proliferation is the key element affecting bone health [12], so it is necessary to research on the influences from high glucose and metformin. In this study, CCK8 assay at day 3 and day 7 revealed that high glucose could suppress the proliferation of osteoblasts, while metformin could attenuate this effect. In addition, the protection of metformin seems independent of the glucose level. The mechanisms of how high glucose disturbs the proliferation and how metformin corrects it could be very complicated and have not yet been elucidated completely. Reactive oxygen species (ROS) system, apoptosis, AMP-activated protein kinase (AMPK), and osteogenic genes are all reported to perform somehow in these procedures [13, 14].

Matrix metalloproteinases (MMPs) are able to degenerate type I collagen, activating bone absorption [10]. Some of the matrix metalloproteinases can augment the expression of the osteogenic genes such as osterix, type 1 collagen, alkaline phosphatase, and osteocalcin [15]. There are few reports about how metformin affects matrix metalloproteinases in osteoblast under high glucose. In our result, mRNA and protein levels of Col*α*, MMP1, and MMP2 were affected by metformin rather than glucose concentration. And Col1*α* seemed to be controlled by metformin at the gene transcription level, aside from the well-known enzyme-substrate level.

It was reported that high glucose (30 mmol/L) increases the expression of MMP2 [16]; and MMP1 expression in peripheral blood was found to be elevated in chronic diabetic patients [17]. However, the elevation of MMP1/2 was not observed in our experiment. The difference may be explained by the relatively moderate glucose level (16.7 mmol/L) and short term of treatment in our experiment. At least in the experiment conditions of our study, aside from those osteogenic genes, MMPs were affected by metformin rather than the glucose level. Whether metformin attenuates the suppression of proliferation through MMPs remains to be determined.

ALP has been widely recognized as one of the most important early marker for osteoblast differentiation [18]. So in this study, ALP activity was used as the indicator of osteoblast differentiation and maturation. Our result showed that high glucose could significantly suppress ALP activity at days 6, 12, and 18, while metformin could rescue them completely at day 6, partly at day 12, but not at day 18. This result implied that the metformin functioned mainly at earlier stage and not in later stage. One of the possible explanations for this interesting phenomenon might be that the osteoblasts on their early stage of differentiation could be more sensitive to chemical stimulations. More work should be done to confirm it in the future.

OPG and RANKL are two important factors produced by osteoblasts, affecting the activation of osteoclasts, so as to function on the protection or absorption of the bone tissues, respectively [19]. So the ratio of OPG/RANKL could be one mark for the balance of the bones [20]. High glucose, some hormones, and lots of chemical compounds are believed to influence the osteoblasts through the OPG/RANKL/RANK system [21–23]. In our studies, high glucose (16.7 mmol/L) and metformin (300 *μ*mol/L) mainly affected the expression of OPG rather than RANKL. In this study, we did experiments on the glucose level of 16.7 mmol/L, which is a relatively moderate glucose level. Future studies with much higher glucose levels might have different results. At least our study indicated that a not-so-high glucose level was already enough to cause a decrease on the ratio of OPG/RANKL, and metformin was a cure to correct it.

## 5. Conclusion

Metformin, a former antidiabetic drug, can protect the MG63 osteoblast-like cells from the harm of high glucose, which not only inhibited the proliferation of the osteoblasts but also decreased the expression of osteogenic genes, for example, Col1*α*, OCN, and OPG. Metformin can inhibit the expression of MMP1 and MMP2, while promoting the expression of Col1*α*, OPG, and OCN. The most possible time course of metformin influencing the osteoblasts is on the early stage of cell proliferation.

## Figures and Tables

**Table 1:** Primers used in the PCR.

Gene	Sense (5′-3′)	Antisense (5′-3′)
Col I	TGTTCAGCTTTGTGGACCTC	CTTGGTCTCGTCACAGATCA
OCN	ATGAGAGCCCTCACACTCCTC	CTAGACCGGGCCGTAGAAGCG
OPG	AGTG GGAG CAGA AGAC ATTG	ATTG GACC TGGT TACC TATC
RANKL	GCGT CGCC CTGT TCTT CTAT	TTGG TGCT TCCT CCTT TCAT
MMP-1	CTCGCTGGGAGCAAACACTG	TGATGTCTGCTTGACCCTCAGAG
MMP-2	GAATGAATACTGGATCTACTCA	TTGTCTCCAGCAAAGATGTATGTC
